# Effect of Phytate (InsP6) and Other Inositol-Phosphates (InsP5, InsP4, InsP3, InsP2) on Crystallization of Calcium Oxalate, Brushite, and Hydroxyapatite

**DOI:** 10.3390/biom13071061

**Published:** 2023-06-29

**Authors:** Paula Calvó, Antònia Costa-Bauza, Felix Grases

**Affiliations:** Laboratory of Renal Lithiasis Research, University Institute of Health Sciences Research (IUNICS-IdISBa), University of Balearic Islands, 07122 Palma de Mallorca, Spain; paula.calvo@uib.es (P.C.); antonia.costa@uib.es (A.C.-B.)

**Keywords:** renal lithiasis, phytate, lower inositol phosphates, crystallization inhibitors, calcium oxalate, calcium phosphates

## Abstract

Pathological calcifications may consist of calcium oxalate (CaOx), hydroxyapatite (HAP), and brushite (BRU). The objective of this study was to evaluate the effect of phytate (inositol hexakisphosphate, InsP6), InsP6 hydrolysates, and individual lower InsPs (InsP5, InsP4, InsP3, and InsP2) on the crystallization of CaOx, HAP and BRU in artificial urine. All of the lower InsPs seem to inhibit the crystallization of calcium salts in biological fluids, although our in vitro results showed that InsP6 and InsP5 were stronger inhibitors of CaOx crystallization, and InsP5 and InsP4 were stronger inhibitors of BRU crystallization. For the specific in vitro experimental conditions we examined, the InsPs had very weak effects on HAP crystallization, although it is likely that a different mechanism is responsible for HAP crystallization in vivo. For example, calciprotein particles seem to have an important role in the formation of cardiovascular calcifications in vivo. The experimental conditions that we examined partially reproduced the in vivo conditions of CaOx and BRU crystallization, but not the in vivo conditions of HAP crystallization.

## 1. Introduction

Phytate was first discovered in vegetable seeds during the 1850s, its chemical formula (inositol hexakisphosphate, InsP6) was established in the early 1900s, and the first animal experiments assessed the effects of phytate consumption in the 1940s [1]. These early animal experiments supplied high doses of sodium phytate to dogs and found that this led to the development of rickets [2]. These studies led to the belief that the consumption of phytate can cause rickets and a deficiency of certain mineral elements, particularly iron, calcium, and zinc. Importantly, these early studies fed dogs with very large amounts of sodium phytate. Other studies that have also found negative effects of consuming high doses of phytate together with low calcium intake have also used sodium phytate [3]. However, phytate naturally occurs in vegetables at a low level, and mainly as a calcium-magnesium salt that is insoluble in water. Therefore, the effect of consuming a moderate amount of phytate when administered in the form of this salt and with a balanced diet does not significantly affect the absorption of calcium (which is actually incorporated in phytate) or other trace elements, such as copper and zinc. However, the consumption of large amounts of phytate with a very unbalanced diet, as occurs in some South Asian countries, can induce deficiencies of trace elements [1]. In fact, the “Mediterranean diet”, which is rich in legumes and nuts, involves a daily consumption of 1 to 2 g of phytin (calcium-magnesium phytate), and this diet clearly has beneficial health effects. By around the year 2000, researchers confirmed the important health benefits from the moderate consumption of phytate with a balanced diet [1]. Phytate is now well-known as an antioxidant [4] as well as for its ability to prevent the development of kidney stones and other pathological calcifications [5], osteoporosis [6], some types of cancer [7,8], and the formation of glycation end-products in patients with diabetes [9,10].

The activity of phytate and its derivatives as inhibitors of the crystallization of calcium salts must be attributed to the presence of various phosphate groups in its molecule, which can interact with areas of nuclei or crystalline surfaces where calcium is found. In fact, all polyphosphates have the capacity to inhibit the crystallization of calcium salts, with pyrophosphate being the first to be described [11]. Precisely because of their hydrolysis in the gastrointestinal tract, Fleisch designed bisphosphonates [12] to treat calcium lithiasis, although they are currently more commonly used for the treatment of osteoporosis [13]. In this sense, phytate has been shown both in in vitro and in vivo studies to prevent the development of calcium kidney stones [5], as well as other pathological calcifications such as cardiovascular ones [5]. We currently know that the consumption of phytate, either by the action of phytases present in the intestine from the food itself or by the action of phosphatases present in the liver or other tissues, gives rise to the formation of various dephosphorylation products, which, being polyphosphates, must also act as inhibitors, as has already been demonstrated in some cases [14].

A notable difficulty in studies that evaluate the effects of InsP6 and its metabolites is determining the levels of all these substances in biological fluids and tissues [15]. One issue is that phytate and other highly phosphorylated InsPs have a very high capacity to bind metallic elements and calcium, and these elements are present in the systems used for detection and determination. A second issue is that the less phosphorylated InsPs have many isomers, and suitable laboratory standards are often unavailable. Moreover, after an individual consumes InsP6, intestinal phytases (also from the diet) cause phytate dephosphorylation [16,17]. All the InsP6 hydrolysates, many of which are likely in low concentrations, cross the intestine by paracellular transport. Once in the liver or other tissues, alkaline phosphatases [1] dephosphorylate these InsPs. Because of these many possibilities, the body contains a great diversity of InsPs, and its levels are affected by InsP6 consumption and endogenous metabolism.

Pyrophosphate is one of the first described inhibitors of calcium salt crystallization in living organisms [18,19]. The digestive system can hydrolyze pyrophosphate. Fleisch first described the chemical synthesis of bisphosphonates [12]. Bisphosphonates are not hydrolyzed by the digestive system, and they are also absorbed at low doses via the paracellular route and are effective crystallization inhibitors, but they are now better known for their prevention of osteoporosis [20]. In fact, all of these polyphosphates (pyrophosphate, bisphosphonates, InsP6, and InsP6 derivatives) have some activities at the intracellular and extracellular level, although these in vivo activities are complex and many are not well known.

The objective of this study is to evaluate the effect of InsP6, mixtures of InsP6 hydrolysates, and individual lower InsPs (InsP5, InsP4, InsP3, and InsP2) on the crystallization of three compounds that are fundamentally involved in pathological calcifications: calcium oxalate (CaOx), hydroxyapatite (HAP), and brushite (BRU).

## 2. Materials and Methods

### 2.1. Reagents

The synthetic urine components were obtained from PanReac (Barcelona, Spain). Myo-Inositol-1,2,3,5,6-pentaphosphate (1,2,3,5,6-InsP5), myo-Inositol-2,3,5,6-tetraphosphate (2,3,5,6-InsP4), myo-Inositol-1,4,5-triphosphate (1,4,5-InsP3), and myo-Inositol-2,4-diphosphate (2,4-InsP2) isomers were purchased from SiChem (Bremen, Germany). Phytic acid sodium salt hydrate and etidronate were purchased from Sigma-Aldrich (Schnelldorf, Bavaria, Germany). Bisphosphonates, alendronate, pamidronate and ibandronate were purchased from Molekula (Darlington, UK). All solutions were prepared in ultra-pure deionized water from a Milli-Q system.

### 2.2. Preparation of InsP6 Hydrolysates

A phytate stock solution (2 mM) was prepared from phytic acid sodium salt and adjusted to pH 2 using 0.5 M HCl. Duplicate aliquots (5 mL) were kept in a metallic dry bath block heater (Selecta, Barcelona, Spain) at 97 °C for 6, 9, 16, 24, 48, or 72 h to obtain the mixtures of its dephosphorilation products at different hydrolysis time.

### 2.3. Evolution of the Relative Proportion of the InsPs in Hydrolysates

The estimation of the evolution of the relative proportion of the InsPs of hydrolysates during thermal hydrolysis was performed by MS/MS spectrometry and the concentration of inorganic phosphate released was determined.

A Q Exactive Orbitrap high-resolution mass spectrometer equipped with a heated electrospray ionization (HESI) probe (ThermoFisher Scientific, Waltham, MA, USA), operated in a negative ionization mode, was used to evaluate the evolution of the relative proportion of the InsPs in function of the hydrolysis time. The temperature of the ion transfer capillary was set to 320 °C, the spray voltage was set to 2.9 kV in negative mode, and the S-lens RF level was 50 AU. Direct injection of properly diluted samples was performed in the full-scan acquisition mode over a range of 150 to 700 *m*/*z* and a resolution of 140,000.

The amount of inorganic phosphate that was liberated during InsP6 hydrolysis was determined using ammonium molybdate and ascorbic acid (a reducing agent), according to the phosphomolybdate–ascorbic acid method [21], measuring the absorbance of the phosphomolybdous complex at 880 nm.

The consideration of the relative MS/MS peaks of each InsP at the different hydrolysis times together with the corresponding liberated inorganic phosphate concentrations allows the estimation of the evolution of the concentration of each InsP in the hydrolysate samples.

### 2.4. Crystallization Experiments

The effects of phytate and the mixtures of its dephosphorilation products at different hydrolysis times on the crystallization of CaOx, HAP, and BRU in synthetic urine were assessed using a kinetic turbidimetric system, as previously described [14]. This system consisted of a spectrometer equipped with a fiber-optic light-guide measuring cell (AvaSpec-ULS2048CL-EVO, Avantes, Apeldoorn, The Netherlands). Crystallization was assessed at a constant temperature (37 °C) with magnetic stirring (300 rpm). Turbidity was used as an indicator of the induction time of CaOx, BRU, and HAP crystallization, and a longer induction time indicated greater inhibition of crystallization.

The induction time (t_i_) was set as the point at which the absorbance cero line crosses the tangent line of the first straight zone of the absorbance curve vs. time. The effects of tested substances on the induction time for the crystallization of CaOx, BRU, and HAP were expressed as the increment of that time (Δ induction time, Δti) with respect to the induction time of the corresponding control, that is, Δti = induction time − induction time of control.

The synthetic urine solution was prepared by making a fresh mixture of equal volumes of solution A and solution B (Table 1), followed by sonication. The pH of this solution was adjusted to 6.0 for CaOx experiments, 6.5 for BRU experiments, and 7.5 for HAP experiments.

For the CaOx experiments, 200 mL of synthetic urine at pH 6.0 was transferred into a 250 mL crystallization flask placed in a thermostatic bath at 37 °C, and 0.2 mL of InsP6 stock solution or a hydrolyzed mixture was added. When the resulting solution reached a temperature of 37 °C, 2 mL of a sodium oxalate stock solution (5 g/L) was added to induce CaOx crystallization.

For the BRU and HAP experiments, 100 mL of synthetic urine solution A at pH 6.5 or 7.5, respectively, was transferred into a 250 mL crystallization flask placed in a thermostatic bath at 37 °C, and 0.2 mL of InsP6 stock solution or a hydrolyzed mixture was added. When the resulting solution reached a temperature of 37 °C, 100 mL of synthetic urine solution B at 37 °C and pH 6.5 or 7.5, respectively, was added to induce crystallization. All experiments were performed in triplicate.

### 2.5. Structural Analysis of Crystals

The morphological and structural characteristics of the CaOx, BRU, and HAP crystals that formed in synthetic urine in the absence or presence of different InsPs were examined using a scanning electron microscopy system (SEM, Hitachi S-3400N, Tokyo, Japan) coupled with XR energy dispersive microanalysis (Bruker AXS XFlash Detector 4010, Berlin, Germany). Crystals formed during crystallization experiments in the absence and presence of additives were collected at the end of each experiment by passing the solution through a 0.45 µm filter. They were then dried in a desiccator and examined by SEM. The methodology used for SEM consists of placing the crystals on a sample holder with fixation on adhesive conductive copper tape, with no need to cover the sample with gold. FTIR spectra of crystals were also obtained by the KBr pellet method with a Bruker Hyperion IR spectrometer (Bruker, Berlin, Germany) to confirm the crystalline phases identified by SEM.

## 3. Results

**Figure 1:**
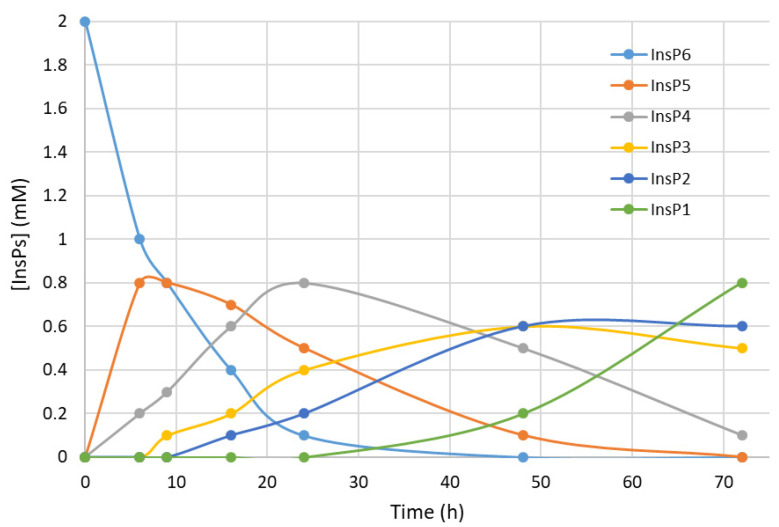
Kinetics of InsP6 hydrolysis in a metallic dry bath block heater at pH 2.0 and 97 °C. Concentrations were estimated from MS/MS measurements and the concentration of inorganic phosphorus and agree with previously published data [22].

We first recorded the evolution of hydrolysates (dephosphorylation products) of InsP6 during thermal hydrolysis in a metallic dry bath block heater at pH 2.0 and 97 °C (Figure 1). The InsP6 level decreased by about 50% and the InsP5 level reached its maximum at about 6 h; InsP4 reached a maximum at about 24 h; and InsP3 and InsP2 reached their maxima at about 48 h. These results coincide with those obtained in previous studies by us [14] and other authors in the same conditions [22].

**Figure 2:**
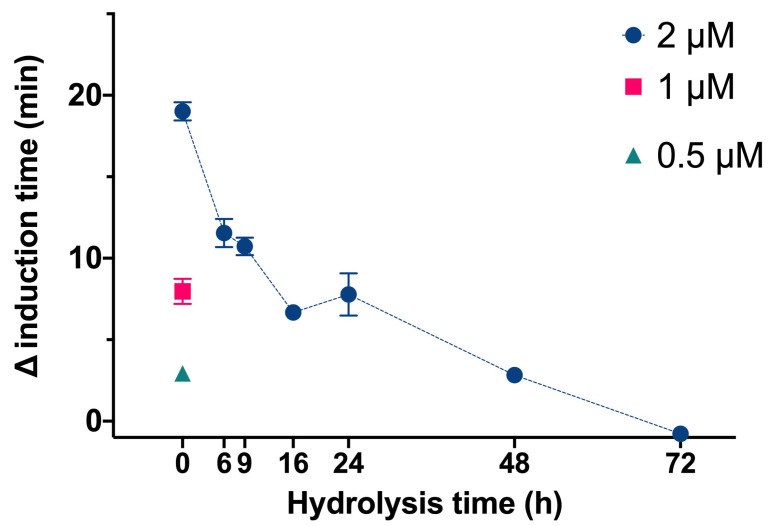
Effect of InsP6 concentration (hydrolysis time: 0 h) and different mixtures of InsP6 hydrolysates (hydrolysis time of 2 µM InsP6: 6 h to 72 h) on the induction time of CaOx crystallization. Values are the means of three experiments ± standard error of mean.

We then determined the effect of InsP6 and the mixtures of its dephosphorilation products at different hydrolysis times on the time needed for CaOx crystallization in artificial urine at pH 6.0 and 37 °C (Figure 2). In this experiment, InsP6 alone (0.5 µM, 1.0 µM, or 2.0 µM) or different mixtures of InsP6 hydrolysates that formed after hydrolysis of 2 µM InsP6 for 6 to 72 h were added, and the evolution of the absorbance of such a solution was recorded in order to determine the induction time of CaOx crystallization. InsP6 had the strongest inhibitory effect, causing a delay of almost 20 min in the induction time of CaOx crystallization compared to that obtained in its absence, while the hydrolysate mixtures collected from 6 h to 72 h had progressively weaker effects. These results are similar to those in previous studies under different conditions [14]. Previous studies demonstrated that although InsP6 had the strongest inhibitory effect on CaOx crystallization (it was also at the highest concentration), a mixture of InsP4 and InsP5 also had significant inhibitory effects.

**Figure 3:**
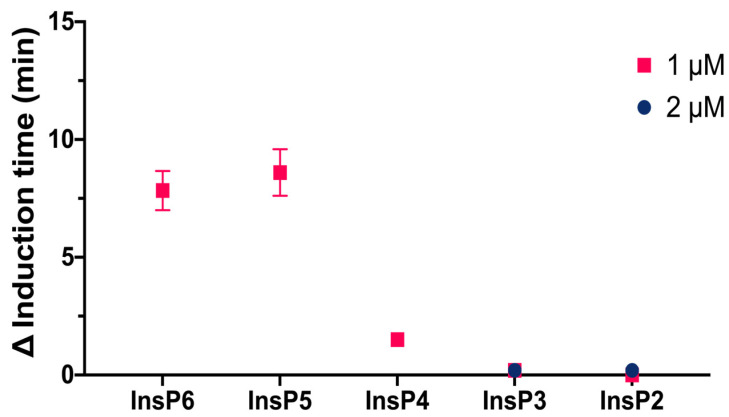
Effect of individual InsPs (InsP6, 1,2,3,5,6-InsP5, 2,3,5,6-InsP4, 1,4,5-InsP3, and 2,4-InsP2 at 1 or 2 µM) on the induction time of CaOx crystallization. Values are the means of three experiments ± standard error of mean.

We then compared the effects of different specific InsPs on the induction time of CaOx crystallization (Figure 3). The results showed that InsP6 and 1,2,3,5,6-InsP5 had similar inhibitory effects, and this effect was greater than that of 2,3,5,6-InsP4, 1,4,5-InsP3, and 2,4-InsP2.

We also compared the effect of four different bisphosphonates at the same concentration to InsPs (2 µM) on the induction time for CaOx crystallization. Under the conditions studied, alendronate, pamidronate, ibandronate, and etidronate had much weaker inhibitory effects (they only caused a delay between 0.6 to 1 min of CaOx crystallization) than InsP6 and its hydrolysates (up to 18 min).

As can be seen in Table 2, both phytate and its dephosphorylated products have weak inhibitory activity against HAP crystallization, even less than bisphosphonates, and it is interesting to observe that this inhibitory capacity increases as the hydrolysis time increases until 48 h, which corresponds mainly to the presence of InsP2 and InsP3, more similar to bisphosphonates.

**Figure 4:**
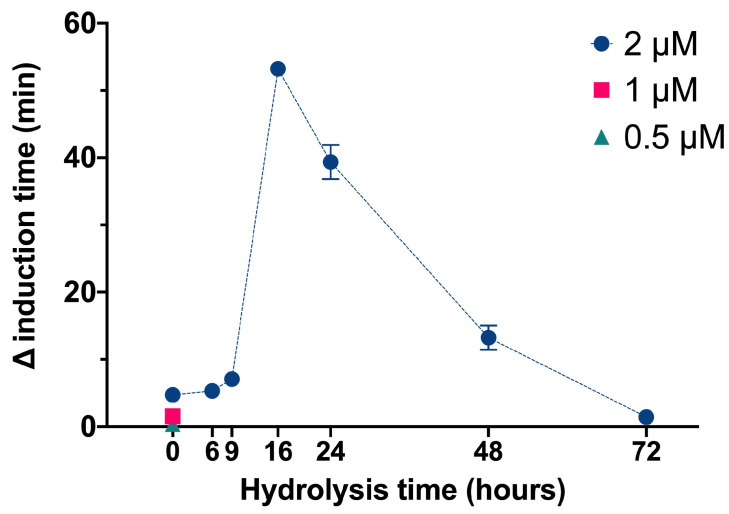
Effect of InsP6 concentration (hydrolysis time: 0 h) and different mixtures of InsP6 hydrolysates (hydrolysis time of 2 µM InsP6: 6 h to 72 h) on the induction time of BRU crystallization. Values are the means of three experiments ± standard error of mean.

**Figure 5:**
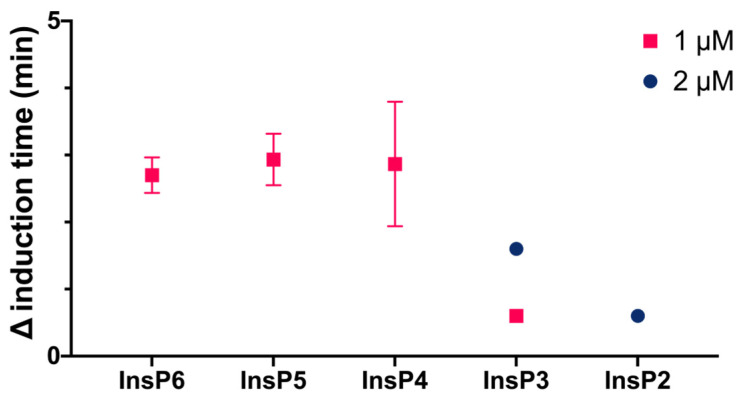
Effect of individual InsPs (InsP6, 1,2,3,5,6-InsP5, 2,3,5,6-InsP4, 1,4,5-InsP3, and 2,4-InsP2 at 1 or 2 µM) on the induction time of BRU crystallization. Values are the means of three experiments ± standard error of mean.

We used the same basic procedures to determine the effect of different InsPs and bisphosphonates on the formation of BRU crystals in artificial urine at pH 6.5 and 37 °C. The results showed that the InsP6 hydrolysates collected at 16 h and 24 h (which were abundant in InsP4 and InsP5) had much stronger inhibitory effects on the crystallization of BRU than InsP6 alone (Figure 4). However, examination of individual InsPs showed that InsP6, 1,2,3,5,6-InsP5, and 2,3,5,6-InsP4 each had about the same inhibitory effects on the crystallization of BRU, and 1,4,5-InsP3 and 2,4-InsP2 had weaker effects (Figure 5).

**Figure 6:**
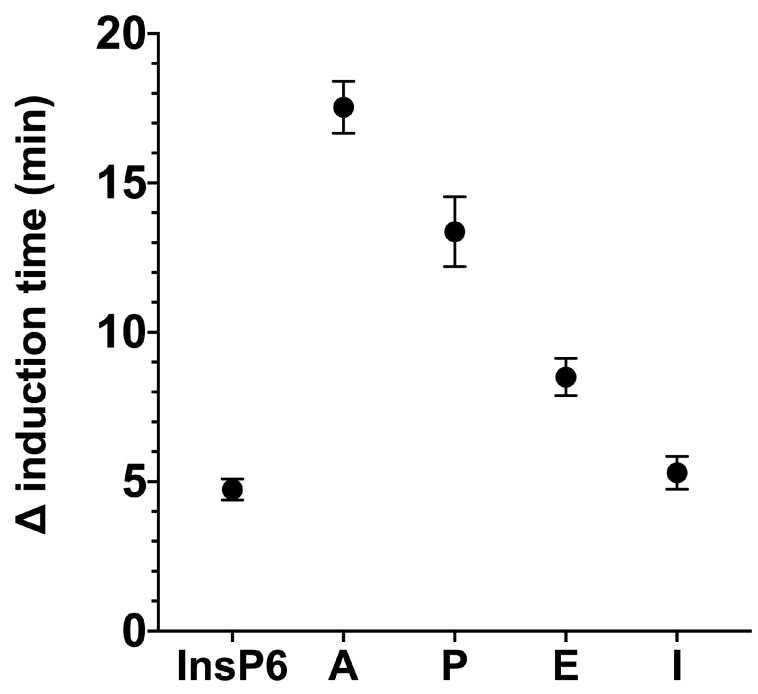
Effect of InsP6 and different bisphosphonates at a concentration of 2 µM on the induction time of BRU crystallization. A: Alendronate; P: Pamidronate; E: Etidronate; I: Ibandronate. Values are the means of three experiments ± standard error of mean.

We then examined the effects of four different bisphosphonates (2 µM) on the inhibition of BRU crystallization relative to 2 µM phytate alone (Figure 6). Alendronate had the greatest inhibition of BRU crystallization, and pamidronate, etidronate, and ibandronate had weaker effects. The effect of ibandronate was similar to that of phytate.

We also determined the effects of four different bisphosphonates and InsP6 and its hydrolysates on the induction time of HAP crystallization (Table 2). These results showed that ibandronate had a remarkable inhibitory effect, followed by alendronate and pamidronate. Moreover, InsP6 and its hydrolysates had very weak inhibitory effects of HAP crystallization under the tested conditions, and the most effective hydrolysates were those collected at 16 h and 48 h (predominantly the lower InsPs).

**Figure 7:**
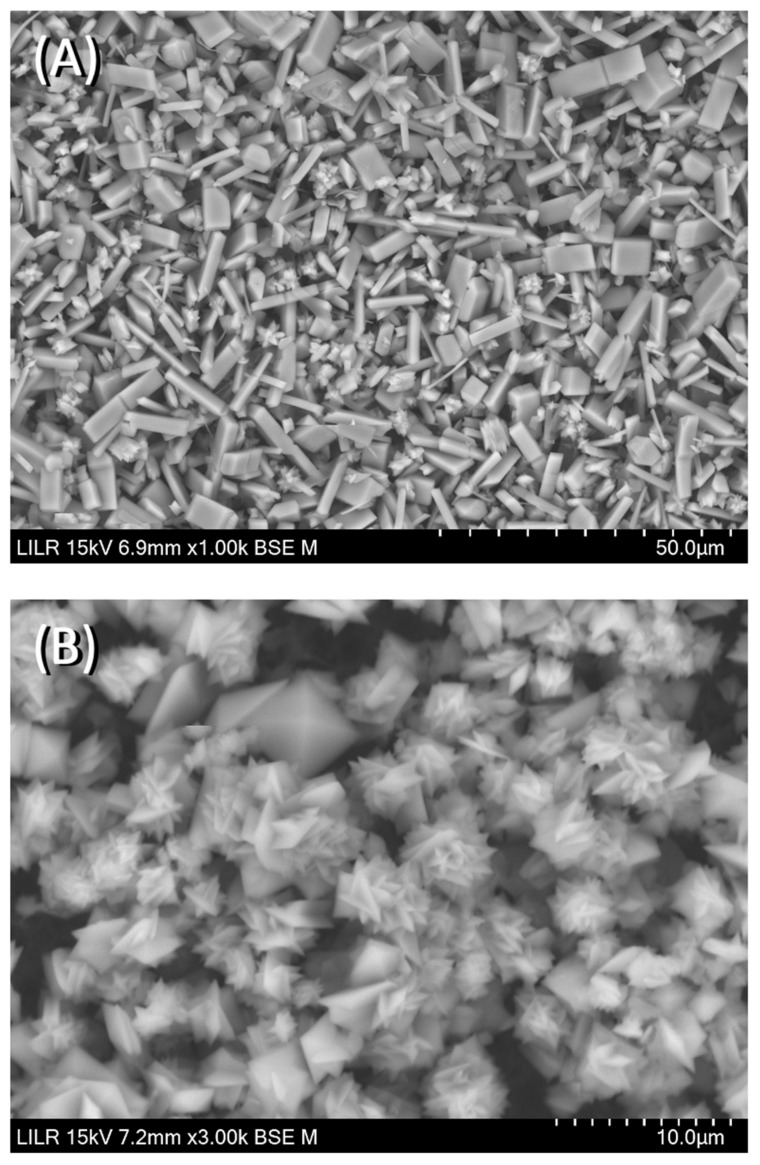
Scanning electron microscopy of CaOx crystals obtained after completion of the turbidimetric assay with (**A**) no phytate (control), (**B**) 2 µM InsP6.

**Figure 8:**
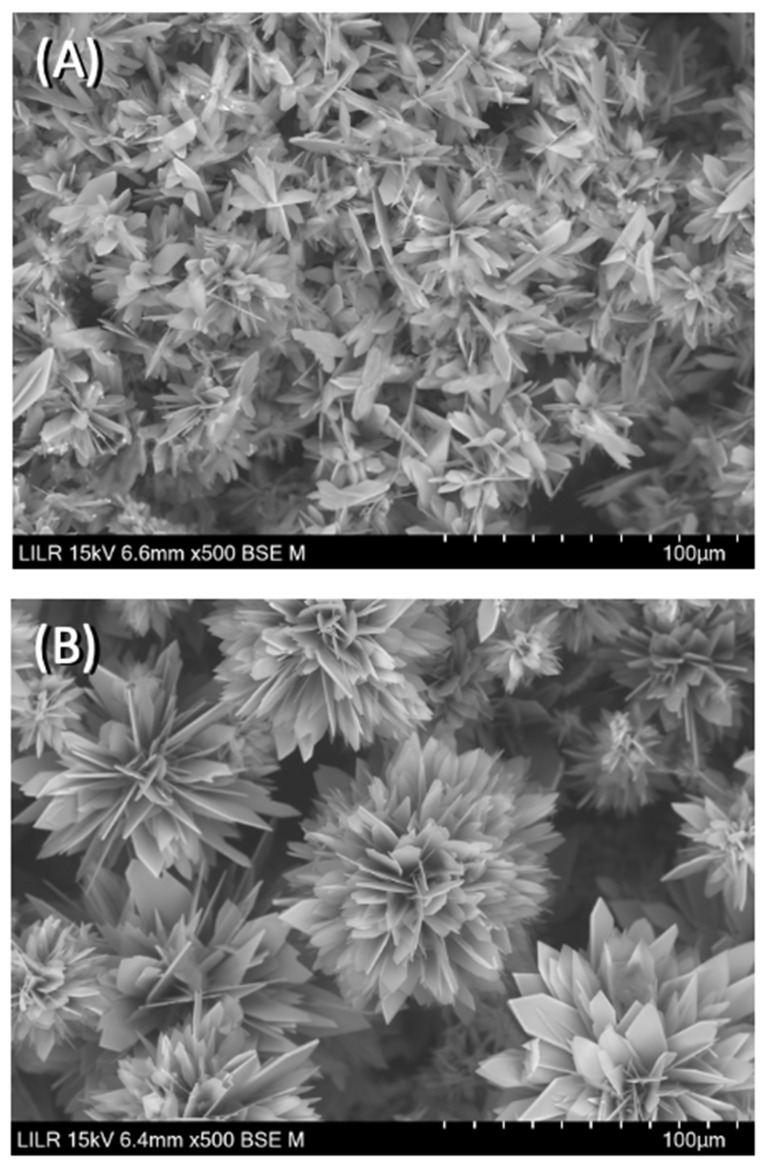
Scanning electron microscopy of BRU crystals obtained after completion of the turbidimetric assay with (**A**) no phytate (control) and (**B**) InsP6 (2 µM) hydrolysate after 16 h.

**Figure 9:**
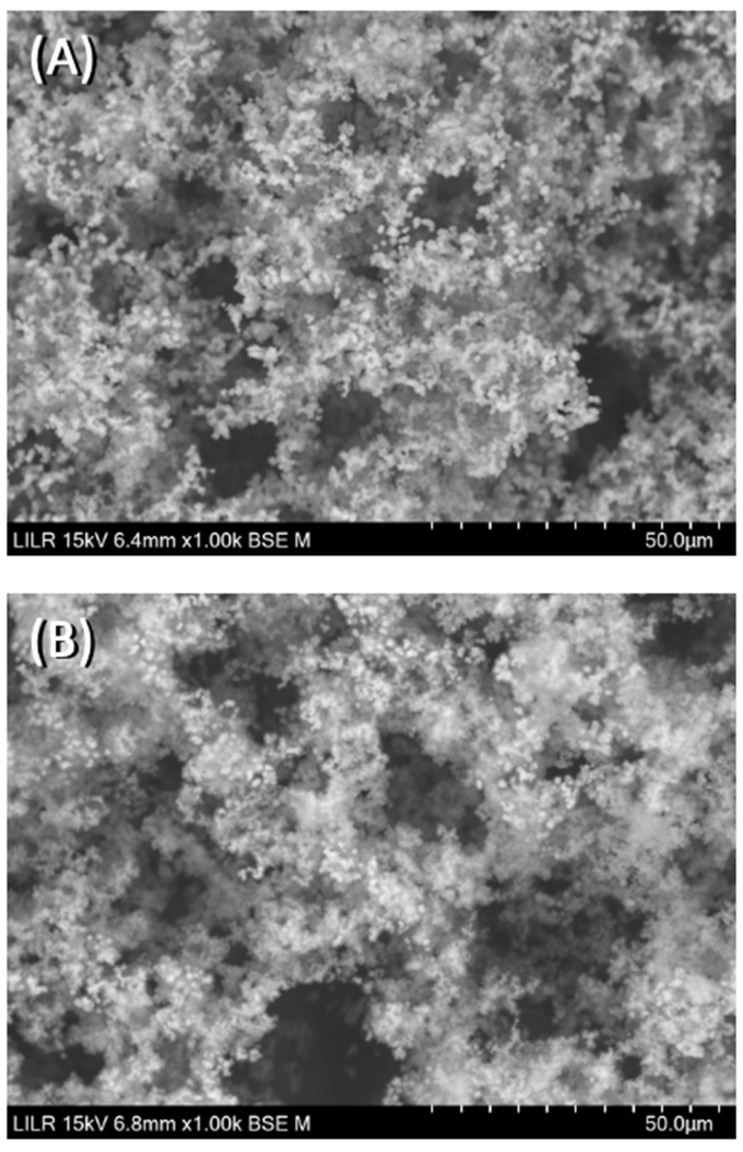
Scanning electron microscopy of HAP spherulites obtained after completion of the turbidimetric assay with (**A**) no phytate (control) and (**B**) InsP6 (2 µM).

We performed scanning electron microscopy to confirm the inhibitory effects of the different treatments. The results showed that CaOx crystallized as a CaOx trihydrate (Figure 7A) in the absence of inhibitors (control), and as a CaOx monohydrate–dihydrate mixture in the presence of InsP5, InsP6 (Figure 7B), and InsP6 hydrolysates that were collected at 6 h. In the presence of the InsP6 hydrolysate obtained after 48 h (mainly containing InsP4, InsP3 and InsP2), the appearance of CaOx trihydrate crystals was observed, and for the InsP6 hydrolysate obtained at 72 h (mainly containing InsP3, InsP2 and InsP1), practically all the formed crystals were CaOx trihydrate. Infrared spectra of crystallized CaOx confirmed the presence of CaOx trihydrate or CaOx monohydrate-dihydrate mixture in each case. The InsP6 hydrolysates that were collected at 16 h and 24 h, InsP5 alone, and InsP4 alone inhibited the formation of BRU crystals and also altered the organization and size of these crystals (Figure 8). In Figure 9, the SEM images of the HAP spherulites obtained in the absence (Figure 9A) and presence (Figure 9B) of InsP6 are shown. As can be seen, these were spheroidal amorphous structures in which individual crystals cannot be distinguished.

## 4. Discussion

The present study extended our previous findings [14] by examining the effect of InsP6 alone, hydrolysates of InsP6, and individual InsPs on the crystallization of CaOx, BRU, and HAP. Our results indicated that the lower InsPs (InsP3 and InsP2) and bisphosphonates had weak effects on CaOx crystallization. However, it must be considered that this inhibition depends on InsP concentration; the more concentrated a species is, the greater the interaction with the nucleation-growth centers, and therefore, the greater its inhibitory effect.

As shown in previous studies [15,23], these lower InsPs are the most abundant InsPs in blood and urine. Because of their higher concentrations, these lower InsPs could have major inhibitory effects in vivo than those observed in the present study, in which low concentrations have been tested.

Our study on InsP6 hydrolysates suggested that InsP4 inhibited CaOx crystallization, but the effect of the pure 2,3,5,6-InsP4 isomer was less than expected. This might be because 2,3,5,6-InsP4 is a weaker inhibitor than the InsP4 isomers that are present in the hydrolysate mixture. Alternatively, it is possible that there are synergistic effects in the mixture of InsP hydrolysates. As noted above, the plasma and urinary levels of InsP4, InsP3, and InsP2 are also much higher than those of InsP6 and InsP5 [15,23], and the inhibition of crystallization is related to the concentration.

Our examination of BRU crystallization showed that the 16 h and 24 h hydrolysates (which were enriched with InsP5 and InsP4) had the strongest effects. Nevertheless, our examination of individual InsPs showed that InsP6, InsP5, and InsP4 had similar effects. As outlined above, these results can be explained by synergistic effects of the multiple InsPs or different InsPs isomers in the hydrolysate.

Interestingly, we found that InsP6 and its hydrolysates had little effect on the crystallization of HAP, and that bisphosphonates (except ibandronate) also had little effect. In contrast, studies with experimental animals and humans showed that the intake of InsP6 significantly reduced HAP calcification [24,25,26]. This may be because the mechanism responsible for the formation of HAP amorphous spherulite deposits is completely different from the mechanism responsible for the formation of CaOx and BRU well-defined crystals [27]. In particular, the formation of a HAP crystal begins with the formation of discrete ion clusters consisting of about 10 to 15 calcium and phosphate ions. These clusters are grouped into larger units (nanoparticles) that form HAP deposits in the presence of appropriate substrates [27,28,29]. For example, calciproteins seem to play an important role in the development of cardiovascular calcifications [30,31]. More precisely, inorganic pyrophosphate (an analogue of bisphosphonates) hinders the nucleation and crystallization of amorphous calcium phosphate and inhibits the growth and maturation of HAP crystals [32]. Therefore, pyrophosphate and polyphosphates affect the evolution of calciprotein particles in vivo in that they can prevent or inhibit calcification. Obviously, because the formation of HAP crystals is strongly dependent on the presence of other compounds, the inhibitory effects we observed in vitro are likely to be very different from those in vivo.

Our results suggest that the effects of InsP6 on experimental animals and humans on crystallization are a consequence of the mixture of the different InsPs. These mixtures of hydrolysates can form in the intestine prior to paracellular absorption (despite the absence of phytases in the human intestine) because certain foods contain phytases. Once in the blood, phytases/phosphatases produced by humans and experimental animals can give rise to lower InsPs. Obviously, we did not demonstrate whether the in vivo effects are due to the presence of certain isomers, differences in the concentrations of different InsPs, or interactions among different InsPs. To answer these questions, it is necessary to use analytical methods that can determine all of these different InsP isomers in biological fluids, and appropriate InsPs standards must be available. At present, most of these standards are not available, and the currently available analytical methods do not allow the precise determination of different InsPs isomers.

Finally, it is necessary to consider that in addition to the direct interference of crystallization by the different InsPs examined here, many other dephosphorylated InsP6 molecules can also indirectly affect crystallization. For example, previous research reported that InsPs can inhibit osteoclastogenesis and mineralization of osteoblasts [33]. Another recent study showed that the intake of InsP6 by menopausal women with hypercalciuria significantly decreased the elimination of urinary calcium [34]. In these cases, phytate or its derivatives do not act as crystallization inhibitors, but due to their activity at the cellular level, the elimination of urinary calcium decreases, so the supersaturation of calcium salts also decreases, and as a consequence their ability to crystallize.

The major limitations of this study are that we did not determine the specific InsPs isomers that formed during the thermal hydrolysis of InsP6, and we did not test the effect of these different isomers. This is simply because these isomers are not readily available or are extremely expensive.

## 5. Conclusions

Although the lower InsPs appear to be responsible for the inhibition of the crystallization of calcium salts in vivo, our in vitro studies showed that the higher InsPs (InsP6 and InsP5) were the most effective in inhibiting CaOx crystallization and that InsP5 and InsP4 were the most effective in inhibiting BRU crystallization. For the specific in vitro experimental conditions of the present study, the InsPs had very weak effects on HAP crystallization. HAP deposits seem to form by a mechanism totally different to the formation of well-defined crystals, depending on the conditions of each case. The in vitro conditions that we examined do not correspond exactly to the in vivo conditions in which HAP deposits are usually formed.

## Figures and Tables

**Table 1:** Composition of synthetic urine.

Solution A		Solution B	
Na_2_SO_4_·10H_2_O	6.23 g/L	NaH_2_PO_4_·2H_2_O	2.41 g/L for CaOx and HAP 4.06 g/L for BRU
MgSO_4_·7H_2_O	1.46 g/L	Na_2_HPO_4_·12H_2_O	5.6 g/L for CaOx and HAP 9.31 g/L for BRU
NH_4_Cl	4.64 g/L	NaCl	13.05 g/L
KCl	12.13 g/L		
CaCl_2_	5 mM for CaOx 4.25 mM for BRU 3 mM for HAP		

**Table 2:** Effect of different bisphosphonates and of phytate and phytate hydrolysis products on the time needed for HAP crystallization. All values are the means of three experiments ± standard error of mean. Δti = induction time–induction time of control.

	Δti (min)
Bisphosphonates	
Alendronate (2 µM)	6.95 ± 0.07
Pamidronate (2 µM)	4.10 ± 0.12
Ibandronate (2 µM)	13.67 ± 1.47
Phytate and Hydrolysis Products	
Phytate (2 µM), nonhydrolyzed	0 ± 0
Phytate (2 µM), 6 h hydrolysis	0 ± 0
Phytate (2 µM), 9 h hydrolysis	0.70 ± 0.06
Phytate (2 µM), 16 h hydrolysis	1.28 ± 0.24
Phytate (2 µM), 24 h hydrolysis	1.90 ± 0.00
Phytate (2 µM), 48 h hydrolysis	1.88 ± 0.28
Phytate (2 µM), 72 h hydrolysis	0.10 ± 0.10

## Data Availability

The data that support the findings of this study are available from the corresponding author [F.G.], upon reasonable request.
